# Editorial

**Published:** 1861-11

**Authors:** 


Scurvy, according to Dr. Rawson, Surgeon to 5th Iowa Regiment,— has already begun to appear among the Missouri troops, in the form of swollen legs, purpura hæmorrhagica, debility, etc.,—the result of a two months' diet of salt meat or bacon, hard bread or flour, very few potatoes and no other vegetables. Some measures should be at once taken to furnish a supply of anti-scrobutics, if it is impossible to procure better rations.

### Editorial.

Medical Department of Lind University.—The regular annual college term in this institution, commenced on Monday, the 4th of October. The introductory lecture was delivered by Prof. E. Andrews, to a full and appreciative audience. Of the lecture itself, we will not speak, as the reader will find it in full in the present number of the Examiner. The term is now fairly in progress, and the courses of instructions, in all departments, going on with the regularity of clock-work. The regular matriculants already number about *sixty*, which shows a most gratifying increase over the number in attendance last year, and clearly indicates the steadily increasing confidence of the profession in the stability of the institution, as well as in the correctness of its plan of instruction. But what is of much more importance, in our estimation, than mere members, is the fact that every member of the present class, has received a good preliminary, and general education, and has entered upon the study of medicine, with habits of industry and mental discipline, coupled with a determination to study. To such a class, it is a pleasure to lecture. When we remember that the present is only the third annual course of lectures since this department of the University was founded; that the present class is double the number of the first; that the institution already possesses a museum of near 7,000 specimens and preparations chiefly anatomical and pathological, a new and ample chemical apparatus and laboratory, and a library of 700 volumes, without one dollar of indebtedness; and that the plans are in active progress for having in readiness a new and permanent college building, at the opening of the next annual session, we see abundant reason for congratulating the

Faculty, and the friends of the school, on its present condition, and fair prospects for the future.

Local Medical Societies.—As we intimated in the October number of the Examiner, the local medical societies in this city have taken a new start, and their meetings are held punctually and well attended. The Chicago Academy of Medicine, holds its regular meetings on the first Friday evening of each month,—and the Chicago Medical Society holds its meetings every Friday evening, except the first one, in each month. We are glad to see, not only the increased attendance, but the greatly increased attention to the subjects chosen for discussion, and the preparation of papers on the part of the members, generally. The profession in this city, contains material for as profitable and interesting social and scientific unions, as exist in any other city.

Communications are at hand, and will receive due attention, —from Ashbel Woodward, M. D., President of the Conn. Med. Society, on *Chloroform in Catalepsy;* from Dr. E. L. Holmes, Surgeon to the Chicago Eye and Ear Infirmary, on *Extirpation of the Eye;* from S. W. Wallace, M. D., of Carthage, Wis., *a Case of Protracted Chloroformic Anæsthesia;* from M. O. Heydock, M. D., of Chicago, on *The Therapeutical Uses of Muriate of Ammonia;* from a New York correspondent, on the opening of the New York Ophthalmic School; and from other correspondents and contributors, all of which we gratefully acknowledge.

Close of the Volume.—The December number will end the second volume of the Examiner. Of the prospects and intentions for the future, however, we do not now propose to speak; but merely allude to the fact, in order to call the attention of our subscribers to the renewal of their subscriptions. The coming number will contain the first of a series of papers on *Ovarian Tumors*, from the pen of Wm. H. Byford, M. D., Professor of Obstetrics and Diseases of Women and Children; also, the Index to the volume, and a number of favors from correspondents and contributors, unfortunately crowded out.

Rush Medical College.—The commencement of the regular college term in this institution, took place on Wednesday, the 16th day of October. The general introductory was given by Prof. DeLaskie Miller. Not being present, we cannot speak definitely, either of the lecture or of the audience, but we presume both were creditable to the institution. The number of students indicates a fair degree of prosperity.

